# Supplementary material for: Molecular Defense Response of Bursaphelenchus xylophilus to the Nematophagous Fungus Arthrobotrys robusta
Source: Cells. 2023 Feb 8;12(4):543. doi: 10.3390/cells12040543 (PMC9953903; doi:10.3390/cells12040543)
Supplement: Supplementary file 1 [file cells-12-00543-s001.zip › Supplementary data.pdf]

Table S1. The primers for RT-qPCR.

Wormbase ID	Forward / Reverse	Primer Sequence
BXYJ_LOCUS5276	BXYJ_LOCUS5276qF	CGCTGGAGTGGTCGTTAAGTTG
	BXYJ_LOCUS5276qR	CGCCGACACATCTCACAGTTG
BXYJ_LOCUS12540	BXYJ_LOCUS12540qF	AGCAACTCCATCTCAGCGAACA
	BXYJ_LOCUS12540qR	CTCCGTCCGATTGTCCACCAT
BXYJ_LOCUS5668	BXYJ_LOCUS5668qF	AAGTATTTCCCGTTGATCGAG
	BXYJ_LOCUS5668qR	TCCCAATTTCTCAGTGTAATCC
BXYJ_LOCUS5675	BXYJ_LOCUS5675qF	TCCCACCTATGCCGACCTT
	BXYJ_LOCUS5675qR	CTCCACTCTCTTCAGATGCTCC
BXYJ_LOCUS2907	BXYJ_LOCUS2907qF	TTATACCCGAACAAACAGGTC
	BXYJ_LOCUS2907qR	ATCTTTCCCGTTCAGTCCG
BXYJ_LOCUS15290	BXYJ_LOCUS15290qF	AGCAATATTTTCCACGCCACA
	BXYJ_LOCUS15290qR	AGAGCACAACGGAACACAAT
BXYJ_LOCUS5680	BXYJ_LOCUS5680qF	AGAAGCGGCACATATTGACCA
	BXYJ_LOCUS5680qR	TTTTCGTCCTGCGTGAATCCC
BXYJ_LOCUS5679	BXYJ_LOCUS5679qF	TCAAAGACACCAGAGTTGCAC
	BXYJ_LOCUS5679qR	TCACACAACGGCTTCCCATC

Table S2. Statistics analysis of RNA sequencing data.

Samples	Raw Data	Clean Data (%)	Raw Data(bp)	Q20 (%)	Q30 (%)	GC (%)	Total Mapped (%)
CK_1	43,381,914	43,316,032 (99.85%)	6,462,678,400	98.16%	94.51%	47.00%	93.79%
CK_2	44,770,464	44,688,496 (99.82%)	6,674,427,728	98.03%	94.18%	47.22%	93.94%
T24_1	45,583,484	45,505,938 (99.83%)	6,781,176,089	98.14%	94.52%	47.39%	90.56%
T24_2	44,691,628	44,612,332 (99.82%)	6,647,868,122	98.05%	94.31%	47.28%	92.44%
T30_1	42,117,894	42,041,160 (99.82%)	6,271,299,608	97.89%	93.84%	47.07%	90.63%
T30_2	44,120,688	44,052,140 (99.84%)	6,566,560,915	98.14%	94.50%	47.10%	91.52%
T36_1	44,841,800	44,757,468 (99.81%)	6,673,663,935	97.86%	93.78%	47.12%	90.12%
T36_2	46,830,432	46,736,896 (99.80%)	6,972,546,638	97.97%	94.09%	47.17%	90.49%
Total	44,0107,102	439,346,946	65,522,186,643				

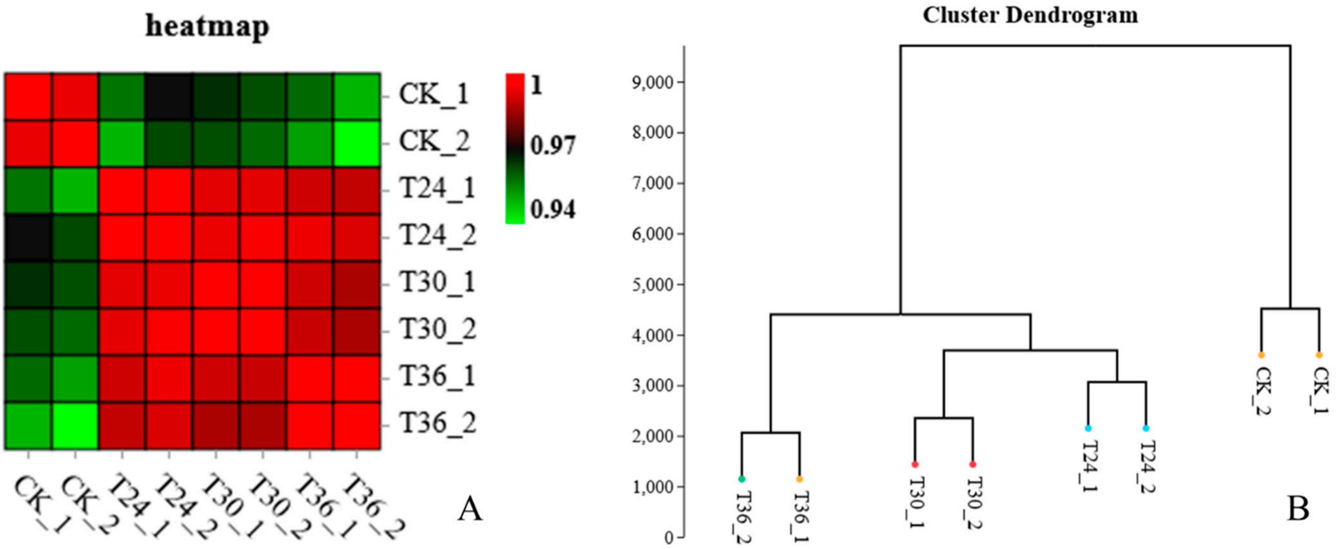

Figure S1. General transcription patterns of all samples. (A). Pearson correlation between controlled and treated nematodes. (B). Clustering of the biological duplicates for each RNA sequencing condition reveals conserved alignment between replicates.

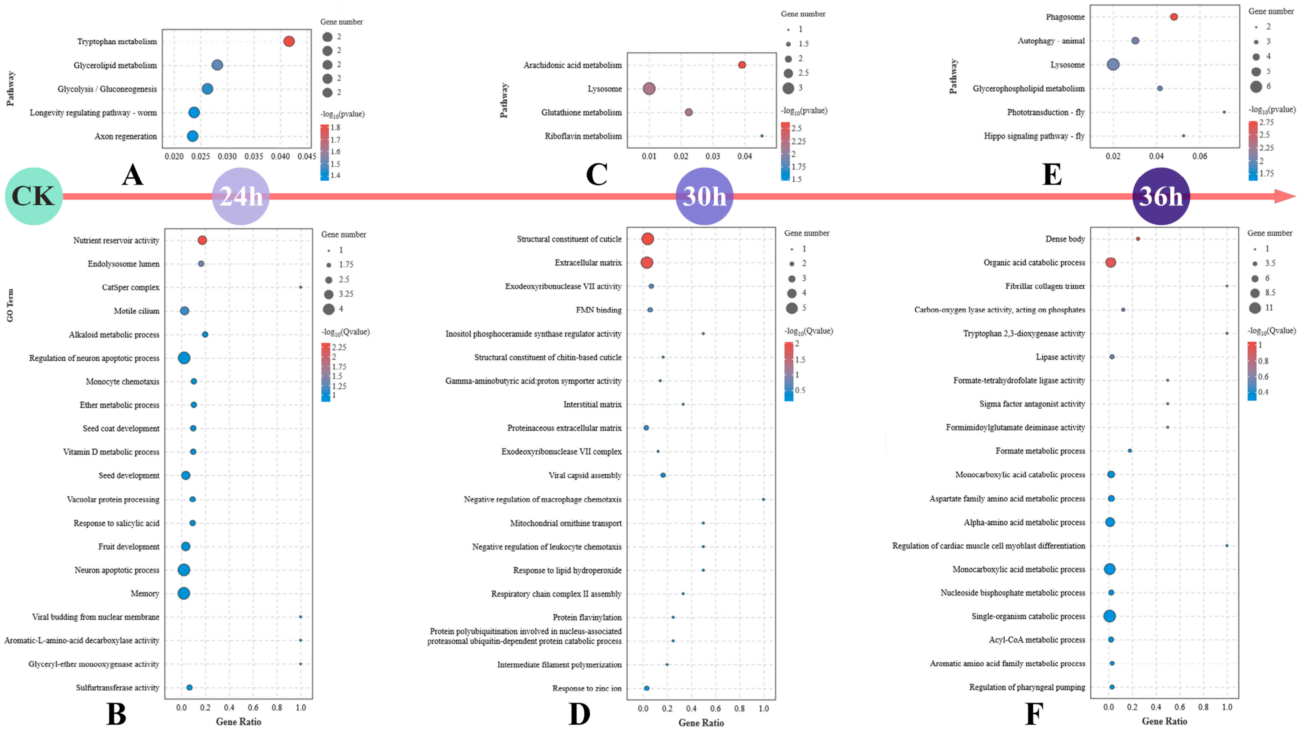

Figure S2. Up-regulated DEGs were identical when PWNs were exposed to *A. robusta*. Up-regulated DEGs were identical when PWNs were exposed to *A. robusta* for 24 h by KEGG (A) and GO analysis; (B) Up-regulated DEGs were identical when PWNs were exposed to *A. robusta* for 30 h by KEGG (C) and GO analysis (D); Up-regulated DEGs were identical when PWNs were exposed to *A. robusta* for 36 h by KEGG (E) and GO analysis (F).
